# Systematic review of CMTX1 patients with episodic neurological dysfunction

**DOI:** 10.1002/acn3.51271

**Published:** 2020-12-12

**Authors:** Dandan Tian, Yating Zhao, Ruixia Zhu, Qu Li, Xu Liu

**Affiliations:** ^1^ Department of Neurology First Affiliated Hospital of China Medical University Shenyang, Liaoning China

**Keywords:** CMTX1, *GJB1*, connexin32, episodic neurological dysfunction, reversible white matter lesion

## Abstract

**Objective:**

X‐linked Charcot‐Marie‐Tooth type 1 (CMTX1) is an inherited peripheral neuropathy caused by mutations in the gap junction beta 1 (*GJB1*) gene, which encodes the connexin32 protein. A small number of patients with *GJB1* mutations present with episodic neurological dysfunction and reversible white matter lesions, which has not been adequately reported. Here, we aim to enable clinicians to further understand this particular situation through systematically reviewing all published relevant cases.

**Methods:**

We conducted a comprehensive search of the PubMed electronic database for medical literature relevant to CMTX1 patients with episodic neurological dysfunction and then fully analyzed the general information, clinical manifestations, and characteristics of magnetic resonance imaging (MRI), cerebrospinal fluid (CSF) analysis, and nerve conduction study (NCS).

**Results:**

We identified 47 cases of CMTX1 associated with episodic central nervous system (CNS) dysfunction from 38 publications. CMTX1 patients experienced episodic CNS deficits at a young age, ranging from infancy to 26 years, and 45 (95.7%) of them were male. The CNS symptoms manifested as facial, lingual, or limb weakness in 44 (93.6%), dysarthria or dysphagia in 39 (83.0%), facial or limb numbness in 15 (31.9%), and ataxia in 10 (21.3%) patients. The duration of episodic symptoms ranged from 3 minutes to 6 months. Thirty (63.8%) CMTX1 cases have reported obvious predisposing factors, among which the most common factors were infection or fever (27.7%), travel to high altitude (12.8%), and intensive exercise (8.5%). As for brain MRI, most abnormal signals were found in bilateral deep white matter (88.9%) and corpus callosum (80.0%). In addition, most of the NCS results were abnormal, including prolonged latency, reduced amplitude, and slowed conduction velocity. The motor nerve conduction velocity (MNCV) of median nerve was the most detectable and valuable, ranging from 25 to 45 m/s.

**Interpretation:**

We have reported the most comprehensive summary of the demographic and clinical profile from 47 CMTX1 patients with episodic CNS deficits and provided new insight into the phenotype spectrum of CMTX1. We hope that our study can help clinicians make early diagnosis and implement the best prevention and treatment strategies for CMTX1 patients with episodic CNS deficits.

## Introduction

Charcot‐Marie‐Tooth (CMT) is widely known as a hereditary neurological disease that mainly affects the motor and sensory fibers of the peripheral nervous system (PNS).^1^ In CMT, X‐linked Charcot‐Marie‐Tooth type 1 (CMTX1) is the second most common form, accounting for 6.5% of all CMTs in a large international series and 10.2% and 10.8% in two important reference centers.^2^, ^3^ Decades of studies have demonstrated that CMTX1 is attributable to mutations in the gap junction beta 1 (*GJB1*) gene on chromosome Xq13.1, which is responsible for encoding the gap junction protein called connexin32 (Cx32).^4^, ^5^ Also, CMTX1 is considered to have a characteristic of X‐linked dominant inheritance without male‐to‐male transmission within kindred.^6^ Most of the affected patients have onset in the first 2 decades of life, with typical manifestations of slowly progressive distal muscle atrophy and weakness, hyporeflexia or areflexia, sensory impairment, and foot deformities such as pes cavus and hammer toes.^7^, ^8^

Although only peripheral neuropathy is present in most CMTX1 patients, episodic neurological dysfunction has been found in a few CMTX1 patients.^9^ In 1998, Panas et al. first reported two cases of central nervous system (CNS) deficits including episodic limb weakness, dysarthria, and dysphagia, which were related to *GJB1* mutation (c.164C > T, p.Ile55Thr).^10^ Since then, more and more clinicians have recognized the special feature of CMTX1 through case reports, but it remains an infrequent occurrence, so collecting information on all published cases is very important. As no systematic review has been performed investigating the characteristics of CMTX1 patients with episodic neurological dysfunction yet, we comprehensively analyzed the general information, clinical manifestations, and the characteristics of magnetic resonance imaging (MRI), cerebrospinal fluid (CSF) analysis, and nerve conduction study (NCS) in all published cases, aiming to pool clinical data to enable clinicians to further understand this particular situation.

## Methods

### Database and search strategies

**Figure 1:**
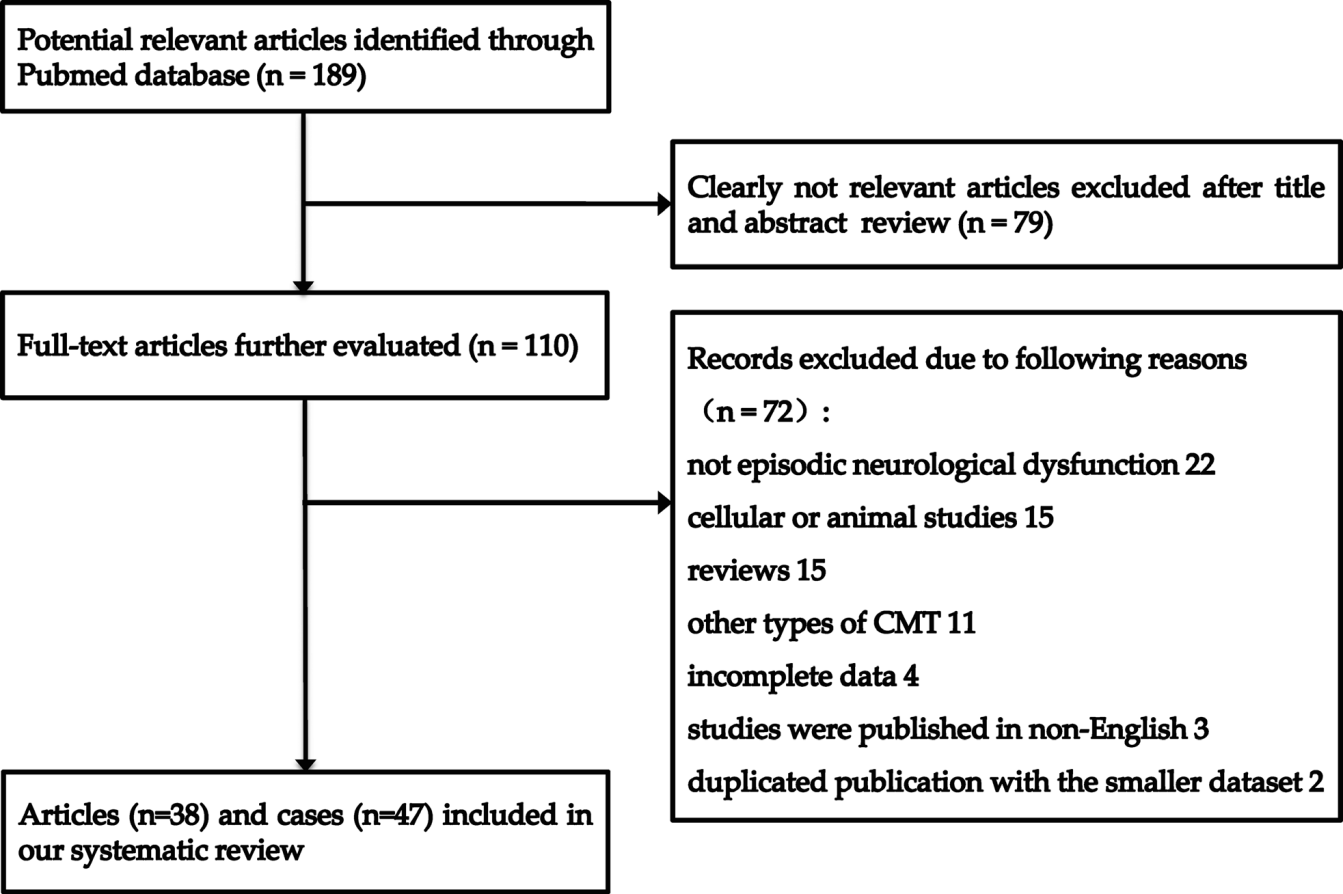
Flow chart depicting our literature search and study selection for the systematic review.

Through a comprehensive literature search on the PubMed electronic database, the articles related to CMTX1 patients with episodic neurological dysfunction were retrieved, and the case reports were systematically collected. The search terms were: CMTX/X‐linked Charcot‐Marie‐Tooth/CMTX1/connexin32/*GJB1* and white matter/leukoencephalopathy /central nervous system/episodic/recurrent/transient/attack/stroke. The screening process of the articles is shown in Figure 1. The latest search was performed on 1 October 2020.

### Inclusion and exclusion criteria

Two researchers independently searched the literature. The inclusion criteria for selected studies were as follows: (1) the case reports of CMTX1 patients with episodic neurological dysfunction; (2) the reports published in English; (3) extractable information in each article. The exclusion criteria met one of the following items: (1) duplicated publication of the smaller dataset; (2) without extractable data; (3) review or abstract. The divergent parts were discussed and agreed.

### Data extraction and classification

For each eligible case report, the following information is extracted and recorded, including the first author's name, publication year, region, demographic characteristics, family history, genetic mutation, clinical manifestations, the results of MRI, CSF analysis, and NCS. In order to classify the clinical factors of CMTX1 patients, some of the above information was further broken down as follows:

1. The region of the patients was drawn from “Asia” (including China/Japan/Korea/India/Turkey), “North America” (including the USA), “Europe” (including Italy/UK/Greece/Germany/Netherlands/Cyprus), and “Oceania” (including Australia).
2. The CNS manifestations were classified as “facial, lingual, or limb weakness”, “dysarthria or dysphagia”, “facial or limb numbness”, “ataxia”, “aphasia”, “pyramidal sign”, and “others”.
3. The PNS examinations were sorted into “areflexia or hyporeflexia”, “pes cavus or hammer toes”, “muscle atrophy or weakness in distal limb”, and “absent or diminished sense”.
4. Predisposing factors were typed into “infection or fever”, “travel to high altitude”, “intensive exercise”, “trauma”, “poor sleep”, “puerperium”, “hyperventilation”, and “prolonged sun exposure”.
5. The MRI lesions can be divided into “bilateral deep white matter”, “corpus callosum”, “splenium of corpus callosum”, “parieto‐occipital regions”, “posterior limbs of internal capsule”, “cerebellar peduncles”, and “others”.
6. Cerebrospinal fluid results were classified into “normal”, “elevated protein levels”, and “elevated white blood cells”.
7. The parameters from NCS including “distal latency”, “compound muscle action potential (CAMP) amplitude”, “motor nerve conduction velocity (MNCV)”, “sensory nerve action potential (SNAP)”, and “sensory nerve conduction velocity (SNCV)” were extracted from median, ulnar, peroneal, tibial, and sural nerves.
8. Treatment was broken down into “corticosteroid and/or intravenous immunoglobulin (IVIg) therapy”, “symptomatic and supportive treatment”, and “without any treatment”.

### Statistical analysis

Continuous variables are expressed as mean ± standard deviation (SD), and categorical variables as percentages. The *t*‐test was used for comparison of continuous variables, while categorical variables were compared by *χ* ^2^ test. All statistical analyses were performed with SPSS Statistics 25 (SPSS Corporation, Chicago, IL). A *P* value of 0.05 was considered statistically significant.

## Results

### Study selection

A total of 189 articles were retrieved, and 79 clearly not relevant articles were excluded by reading titles and abstracts. By reading the full text of the remaining 110 articles, 22 were not related to episodic neurological dysfunction, 15 were cellular or animal studies, 15 were reviews, 11 reported other types of CMT patients, four had incomplete data, three were not published in English, and two were duplicated publications with the smaller dataset. Finally, we identified 47 cases from 38 articles that met the criteria of our systematic review. ^11^, ^12^, ^13^, ^14^, ^15^, ^16^, ^17^, ^18^, ^19^, ^20^, ^21^, ^22^, ^23^, ^24^, ^25^, ^26^, ^27^, ^28^, ^29^, ^30^, ^31^, ^32^, ^33^, ^34^, ^35^, ^36^, ^37^, ^38^, ^39^, ^40^, ^41^, ^42^, ^43^, ^44^, ^45^, ^46^, ^47^, ^48^ The gradual selection and exclusion process of the studies is shown in Figure 1.

### General information of reported cases

A total of 47 cases were selected in the present study. Tables 1 and 2 list the demographic characteristics, family history, and genetic mutations of CMTX1 patients with episodic CNS deficits. All CMTX1 patients experienced episodic CNS deficits at a young age, ranging from infancy to 26 years. Among them, there were 45 (95.7%) male patients and two (4.3%) female patients. As for the geographic information of patients, 16 (34.0%) were drawn from Asia, 15 (31.9%) from North America, 15 (31.9%) from Europe, and one (2.1%) patient from Oceania.

**Table 1:** The demographic characteristics, family history, and genetic mutations in CMTX1 patients with episodic neurological dysfunction.

Patient Number	Age (Age onset)	Gender	Region	Family History	Nucleotide Transition	Amino acid Substitution	1^st^ Author (Year)	Reference
1	38 (11)	Male	Cyprus	Older brother	c.381C > G	p.Ile127Met	Tziakouri, A. (2020)	[11]
2	7 (6)	Male	China	None	c.391C > T	p.Arg107Trp	Niu, J. (2020)	[12]
3	29 (21)	Female	China	None	c.622G > A	p.Glu208Lys	Li, Q. (2020)	[13]
4	28 (14)	Male	China	Maternal relatives	c.‐170T > G	NCR^#^	Luo, S. (2019)	[14]
5	20 (20)	Male	China	Mother, maternal grandfather and cousin	c.380T > C	p.Ile127Thr	Hu, G. (2019)	[15]
6	13 (9)	Male	USA	None	c.227T > C	p.Leu76Pro	Hardy, D. I. (2019)	[16]
7	14 (14)	Male	USA	None	c.425G > A	p.Arg142Gln	Hardy, D. I. (2019)	[16]
8	13 (13)	Male	USA	Mother, brother	c.227T > G	p.Leu76Arg	Santoro, J. D. (2019)	[17]
9	17 (12)	Male	China	Mother, maternal grandfather and aunts, other relatives	c.425G > A	p.Arg142Gln	Liang, Y. (2019)	[18]
10	15 (15)	Male	China	Mother, maternal grandfather, aunt and her daughter	c.563C > T	p.Thr188Ile	Liang, Y. (2019)	[18]
11	18 (18)	Male	China	Mother, younger maternal aunt, grandfather’s brother	c.103G > C	p.Val35Leu	Liang, Y. (2019)	[18]
12	15 (15)	Male	Turkey	None	c.542T > C	p.Val181Ala	Aktan, Z. (2018)	[19]
13	28 (10)	Male	USA	Mother	c.271G > A	p.Val91Met	Nicholso, P. D. (2017)	[20]
14	22 (22)	Male	Greece	None	c.223C > T	p.Arg75Trp	Parissis, D. (2017)	[21]
15	11 (11)	Male	Korea	None	c.283G > A	p.Val95Met	Kim, J. K. (2017)	[22]
16	24 (18)	Male	China	Mother, grandfather, aunt	c.445T > C	p.Phe149Leu	Xie, C. (2016)	[23]
17	20 (13)	Male	USA	Mother, maternal grandfather, and aunt, son of affected aunt	c.467T > G	p.Leu156Arg	Wu, N. (2015).	[24]
18	13 (13)	Male	China	Mother, grandfather	c.490C > T	p.Arg164Trp	Shu, X. M. (2015)	[25]
19	19 (8)	Male	India	Mother, maternal grandfather and male cousins, elder brother	c.425G > A	p.Arg142Gln	Kulkarni, G. B. (2015)	[26]
20	15 (15)	Male	China	Mother, his mother’s mother and sister	c.278T > G	p.Met93Arg	Zhao, Y. (2014)	[27]
21	29 (16)	Male	Italy	Mother, maternal grandmother	c.297_298 insCAA	p.Gln99_His 100insGln	Sagnelli, A. (2014)	[28]
22	12 (12)	Male	USA	Mother	c.98T > A	p.lle33Asn	McKinney, J. L. (2014)	[29]
23	14 (14)	Male	Korea	Mother, maternal grandparents and aunt	c.3G > T	p.Met1Ile	Kim, G. H. (2014)	[30]
24	14 (14)	Male	USA	Mother and half‐brother	c.179G > A	p.Cys60Tyr	Appu, M. (2014)	[31]
25	14 (14)	Male	USA	None	c.260C > G	p.Pro87Leu	Al‐Mateen, M. (2014)	[32]
26	17 (9)	Male	USA	Mother	c.477G > A	p.Val139Met	Al‐Mateen, M. (2014)	[32]
27	17 (14)	Male	China	Mother, maternal grandfather	c.161A > G	p.Asn54Ser	Zhong, L. (2012)	[33]
28	28 (12)	Male	Japan	Brother, sister	c.396G > A	p.Trp132*	Sato, K. (2012)	[34]
29	15 (12)	Male	Japan	Mother	c.397delT	p.Trp133*	Sakaguchi, H. (2011)	[35]
30	11 (11)	Male	UK	Mother	c.196G > A	p.Asp66Asn	U‐King‐Im, J. M. (2011)	[36]
31	21 (21)	Male	UK	Mother, maternal grandfather	c.556G > T	p.Glu186*	Basu, A. (2011)	[37]
32	15 (5)	Male	UK	Mother	c.80T > C	p.Val27Ala	Absoud, M. (2011)	[38]
33	10 (10)	Male	USA	Mother, maternal grandmother, half‐uncle and half‐first cousin	c.65G > A	p.Arg22Gln	Rosser, T. (2010)	[39]
34	14 (14)	Male	Italy	Mother, maternal grandmother and uncles	c.491G > A	p.Arg164Gln	Fusco, C. (2010)	[40]
35	7 (7)	Male	UK	None	c.530T > C	p.Val177Ala	Anand, G. (2010)	[41]
36	10 (10)	Male	Australia	Mother and three additional family members	c.65G > A	p.Arg22Gln	Srinivasan, J. (2008)	[42]
37	13 (13)	Male	USA	Mother, brother, maternal relatives	c.417G > A& c.419C > G	p.Val139Met	Halbrich, M. (2008)	[43]
38	16 (6)	Male	USA	Mother, brother, maternal relatives	c.417G > A& c.419C > G	p.Val139Met	Halbrich, M. (2008)	[43]
39	12 (12)	Male	USA	Mother, maternal grandmother, great‐grandmother, two uncles, aunt and her son	c.285C > T	p.Arg75Trp	Taylor, R. A. (2003)	[44]
40	43 (7)	Female	Germany	Two sons	c.304_306 delGAG	p.102delGlu	Hanemann, C. O. (2003)	[45]
41	12 (10)	Male	Germany	Mother brother	c.304_306 delGAG	p.102delGlu	Hanemann, C. O. (2003)	[45]
42	19 (Infancy)	Male	Germany	Mother brother	c.304_306 delGAG	p.102delGlu	Hanemann, C. O. (2003)	[45]
43	14 (4)	Male	Netherlands	Mother	c.490C > T	p.Arg164Trp	Schelhaas, H. J. (2002)	[46]
44	29 (26)	Male	USA	Maternal relatives	c.424C > T	p.Arg142Trp	Paulson, H. L. (2002)	[47]
45	16 (16)	Male	USA	None	c.565G > A	p.Cys168Tyr	Paulson, H. L. (2002)	[47]
46	21 (10)	Male	Greece	Mother, brother	c.164C > T	p.Ile55Thr	Panas, M. (2001)	[48]
47	19 (12)	Male	Greece	Mother, brother	c.164C > T	p.Ile55Thr	Panas, M. (2001)	[48]

NCR: noncoding region; UA: unavailable.

^#^ The variant c.‐170T > G is located in nerve‐specific promoter P2 region of *GJB1*.

**Table 2:** The clinical manifestations and treatment in CMTX1 patients with episodic neurological dysfunction.

	N (%)
Patients (n)	47
Age onset (years), median (range)	12 (Infancy‐26)
Gender, male : female	45 (95.7) : 2 (4.3)
Family History	
Yes	37/47 (78.7)
No	10/47 (21.3)
Region	
Asia	16/47 (34.0)
North America	15/47 (31.9)
Europe	15/47 (31.9)
Oceania	1/47 (2.1)
CNS Manifestations	
Facial, lingual or limb weakness	44/47 (93.6)
Dysarthria or dysphagia	39/47 (83.0)
Facial or limb numbness	15/47 (31.9)
Ataxia	10/47 (21.3)
Aphasia	5/47 (10.6)
Pyramidal sign	14/47 (29.8)
Predisposing factors	
Infection or fever	13/47 (27.7)
Travel to high altitude	6/47 (12.8)
Intensive exercise	4/47 (8.5)
Trauma	2/47 (4.3)
Poor sleep	2/47 (4.3)
Thyroid malfunction	1/47 (2.1)
Puerperium	1/47 (2.1)
Hyperventilation	1/47 (2.1)
Exposure to strong sunshine	1/47 (2.1)
Time to recovery (range)	3 minutes – 6months
Episode(s)	
Recurrent	39/47 (83.0%)
Single	8/47 (17.0%)
PNS Manifestations	
Hyporeflexia or areflexia	38/45 (84.4)
Pes cavus or hammer toes	31/45 (68.9)
Muscle atrophy or weakness in distal limb	23/45 (51.1)
Diminished or absent sense	19/45 (42.2)
Treatment	
Corticosteroid and/or IVIg therapy	14/27 (51.9)
Symptomatic and supportive treatment	9/27 (33.3)
Without any treatment	4/27 (14.8)

Abbreviations: CNS, central nervous system; IVIg, intravenous immunoglobulin; PNS, peripheral nervous system.

Of the 47 patients we systematically reviewed, 37 (78.7%) had a family history consistent with X‐linked inheritance. All patients presented the data of genetic mutations, including 39 (83.0%) missense mutations, four (8.5%) deletion mutations, three (6.4%) nonsense mutations, one (2.1%) insertion mutation, and one (2.1%) regulatory mutation in the noncoding region of *GJB1*. Moreover, we re‐analyzed the pathogenicity of all the mutations in our systematic review by using gnomAD database, REVEL scores, and ClinVar database, and included case reports and other relevant studies. ^49^, ^50^, ^51^ As shown in Table S1, we found that all the mutations in CMTX1 patients with episodic neurological deficits were pathogenic or likely pathogenic based on ACMG criteria ^52^.

### Clinical manifestations

The clinical manifestations in CMTX1 patients with episodic neurological deficits are presented in Tables 2 and Table S2. The most common CNS symptom was facial, lingual, or limb weakness, which has been reported in 44 cases, accounting for 93.6%. Dysarthria, dysphagia, or both followed closely, with 39 (83.0%) patients reporting this situation. The third and fourth most common symptoms were facial or limb numbness and ataxia. Meanwhile, some patients had relatively rare CNS manifestations, including aphasia, cranial nerve deficits, choreiform movements, dizziness, and lethargy. Among these 47 patients, the duration of episodic symptoms ranged from 3 minutes to 6 months, of which 39 had more than one episode. In addition, during the neurological examination, the pyramidal sign was found in 14 (29.8%) patients. As for predisposing factors for episodic CNS deficits, 30 (63.8%) CMTX1 cases have reported obvious predisposing factors in our systematic review. The CNS symptoms followed an infection or fever in 13 (27.7%), travel to high altitude in six (12.8%), intensive exercise in four (8.5%), trauma in two (4.3%), poor sleep in two (4.3%), thyroid malfunction in one (2.1%), puerperium in one (2.1%), hyperventilation in one (2.1%), and exposure to strong sunshine in one (2.1%) patient. Moreover, the specific predisposing factors for these four patients with intensive exercise included a football training, a workout in the gym, wrestling with his brother, and several days of heavy manual labor. In addition, PNS examinations were documented in 45 CMTX1 patients, of which 38 (84.4%) had hyporeflexia or areflexia in extremities, 31 (68.9%) had pes cavus or hammer toes, and 23 (51.1%) had muscle atrophy or weakness in distal limb. On the opposite, two (4.3%) cases had a normal physical examination of PNS.

### MRI and CSF

The MRI and CSF findings of CMTX1 patients with episodic CNS deficits are shown in Tables 3 and Table S3. Overall, 45 patients underwent MRI examination and 40 (88.9%) had increased T2, fluid‐attenuated inversion recovery (FLAIR), or diffusion‐weighted image (DWI) signals in bilateral deep white matter. There were 36 (80.0%) patients with corpus callosum lesions, of which 26 (72.2%) involved only the splenium. Abnormal MRI signals were also found in parieto‐occipital regions, the posterior limbs of internal capsule, and cerebellar peduncles. During the follow‐up, all MRI abnormalities returned to normal or had obvious improvement, ranging from 9 days to 2 years. In addition, CSF tests were performed in 33 patients. The results showed that 25 (75.8%) were normal, eight (24.2%) had elevated protein levels, and two (6.1%) cases had elevated white blood cells.

**Table 3:** The characteristics of MRI, CSF analysis, and NCS in CMTX1 patients with episodic neurological dysfunction.

	N (%)
MRI lesions	
Bilaterally deep white matter	40/45 (88.9)
Corpus callosum	36/45 (80.0)
Splenium of corpus callosum	26/36 (72.2)
Parieto‐occipital regions	15/45 (33.3)
Posterior limbs of internal capsule	7/45 (15.6)
Cerebellar peduncle	5/45 (11.1)
Time of MRI improvement (range)	9 days – 2 years
CSF	
Normal	25/33 (75.8)
Elevated protein levels	8/33 (24.2)
Elevated white blood cells	2/33 (6.1)
Nerve conduction study: mean ± SD	
Median nerve	
Distal latency (ms)	6.3 ± 2.3
CMAP amplitude (mV)	3.7 ± 3.0
MNCV (m/s)	36.0 ± 6.7
SNAP amplitude (µV)	7.1 ± 7.3
SNCV (m/s)	38.3 ± 4.0
Ulnar nerve	
Distal latency (ms)	5.7 ± 3.0
CMAP amplitude (mV)	7.8 ± 9.9
MNCV (m/s)	36.7 ± 9.7
SNAP amplitude (µV)	4.2 ± 2.0
SNCV (m/s)	38.7 ± 4.8
Peroneal nerve	
Distal latency (ms)	8.0 ± 5.0
CMAP amplitude (mV)	1.1 ± 1.0
MNCV (m/s)	33.3 ± 4.2
Tibial never	
Distal latency (ms)	10.1 ± 5.6
CMAP amplitude (mV)	2.1 ± 2.0
MNCV (m/s)	32.7 ± 6.5
Sural nerve	
SNAP amplitude (µV)	3.6 ± 0.9
SNCV (m/s)	33.9 ± 5.8

Abbreviations: CMAP, compound muscle action potential; CSF, cerebrospinal fluid; MNCV, motor nerve conduction velocity; MRI, magnetic resonance imaging; NCS, nerve conduction study; SD, standard deviation; SNAP, sensory nerve action potential; SNCV, sensory nerve conduction velocity.

### Nerve conduction study

NCS was mentioned in 40 CMTX1 patients with episodic CNS deficits, of which 38 were abnormal and 17 had specific data for extraction (Tables 3 and Table S4). The prolongation of distal latency was observed in all nerves except for ulnar nerve in two cases. The mean ± SD of distal latency in median, ulnar, peroneal, and tibial nerves was 6.3 ± 2.3, 5.7 ± 3.0, 8.0 ± 5.0, and 10.1 ± 5.6 ms, respectively. As for MNCV, it was reduced in all patients and the mean ± SD in median, ulnar, peroneal, and tibial nerves was 36.0 ± 6.7, 36.7 ± 9.7, 33.3 ± 4.2, and 32.7 ± 6.5 m/s, respectively. Among them, the MNCV of median nerve was the most detectable and valuable, ranging from 25 to 45 m/s. Similar to MNCV, most cases had decreased CMAP amplitude compared with normal values. In addition, for median, ulnar, and sural nerves, SNCV and SNAP were observed to decline as well. In total, the results of the NCS revealed a peripheral polyneuropathy involving both motor and sensory fibers with, in most cases, intermediate conduction slowing.

### Clinical treatment

The data on the treatment were available in 27 patients, of which 14 (51.9%) received corticosteroid and/or IVIg therapy, nine (33.3%) received symptomatic and supportive treatment, and four (14.8%) patients did not receive any treatment (Table 2). Impressively, we did not find a statistically significant difference in the recovery time of CNS symptoms in patients who received or did not receive corticosteroid and/or IVIg treatment (*P* = 0.199).

## Discussion

CMTX1 is a hereditary disease involving peripheral nerves with variable clinical phenotypes. In recent years, a few CMTX1 cases with transient stroke‐like symptoms and reversible white matter lesions have been reported. Considering the paucity of this phenotype, the systematic collection of the clinical data for CMTX1 patients with episodic CNS symptoms is of great significance for clinicians to further understand their special features. Although Hu et al. performed a literature review on CMTX1 patients with CNS involvement, only 19 patients with episodic neurological dysfunction were included in less detail.^15^ Therefore, in order to obtain a deeper understanding of this particular phenotype of patients with CMTX1, we performed this comprehensive systematic review of 47 CMTX1 patients with episodic CNS deficits worldwide.

CMTX1 is an X‐linked dominant inheritance mode, which means that hemizygous male patients usually exhibit the full phenotype, while female carriers show variable degrees of peripheral neuropathy and are thought to be protected by CNS dysfunction. According to our results, the obvious gender difference was also found in CMTX1 patients with episodic CNS deficits. Hemizygous men were obviously those affected by transient CNS dysfunction, accounting for 95.7% of all patients. However, the involvement of two heterozygous women is notable, which could be explained as a result of skewed X‐inactivation.^53^, ^54^, ^55^, ^56^ Since the pattern of X‐inactivation varies between different types of tissues or cells, we speculate that X‐inactivation in oligodendrocytes of the CNS may be skewed and in these two women the normal allele was preferentially inactivated while maintaining disease‐causing allele expression, thus leading to episodic CNS deficits.^57^, ^58^, ^59^

As is well known, Cx32 is a transmembrane protein on the oligodendrocyte side of astrocyte‐oligodendrocyte junctions that is involved in the formation of gap junction channels in vertebrates.^60^, ^61^ Mutations in the *GJB1* gene that encodes Cx32 are considered to be the reason for CMTX1. Mechanistically, these mutations may cause Cx32 dysfunction, thereby inhibiting the assembly or pore formation of transmembrane channels, reducing the luminal diameter of the channels, or being more sensitive to acidification‐induced closure.^62^, ^63^, ^64^ Moreover, these fragile channels may lead to temporary functional derangement of gap junctions with increased water content and possibly intramyelinic edema, which can be partially confirmed by such a rapid recovery and abnormal diffusion restriction on the patients' MRI.

Previous cases have shown that in more than half of patients, episodic CNS symptoms occurred under conditions of metabolic stress, including travel to high altitude, intensive exercise, hyperventilation, and infection. For patients with high altitude travel, intensive exercise, or hyperventilation, it is presumed that the increased acidification of the CSF due to hypoxia may result in abnormal channel closure and ultimately dysfunction of gap junctions.^65^ Among patients with infectious factors, the possible mechanism is that the proinflammatory cytokines released during infection may lead to reduced gap junctions between oligodendrocytes and astrocytes.^9^ Besides, we previously reported that a CMTX1 female, during the puerperium, developed recurrent stroke‐like symptoms at 3 weeks after a normal pregnancy and a smooth cesarean section.^13^ It is hypothesized that during the puerperium, the sudden decrease in estrogen and progesterone levels would impede the proliferation and maturation of oligodendrocyte progenitor cells.^66^, ^67^ Thus, CMTX1 patients with fragile gap junctions owing to the mutations of Cx32 may be more vulnerable to these predisposing factors, which may induce transient neurological deficits. Further research is needed to understand these potential predisposing factors on episodic CNS dysfunction so that patients may be advised appropriately.

In the past, clinicians' understanding of the clinical manifestations of CMTX1 patients mainly included progressive distal muscle atrophy and weakness, sensory impairment, hyporeflexia or areflexia, and pes cavus. However, in recent years, several cases have exhibited episodic symptoms of CNS, such as facial, lingual or limb weakness, numbness, dysarthria or dysphagia, and ataxia. Besides, aphasia, cranial nerve deficits, choreiform movements, dizziness, and lethargy have also been reported, and we cannot exclude the diagnosis of CMTX1 if patients experience these rare episodic symptoms. In addition to episodic symptoms, we found that brain MRI abnormalities were one of the critical characteristics for patients with CNS involvement. First, after attack, brain MRI usually showed increased T2/FLAIR/DWI signals in bilateral deep white matter as well as corpus callosum. Moreover, abnormal MRI signals were reversible and more visible in the posterior regions, while subcortical U‐fibers were generally spared. Second, abnormalities of the corpus callosum were impressive, and 26 of 36 cases affected only the splenium. Thus, in addition to seizure, infection, and metabolic disturbance, CMTX1 should be considered as another rare cause of reversible splenial lesion syndrome (RESLES).^68^, ^69^, ^70^ Third, the recovery of T2/FLAIR abnormal signals occurred later than clinical symptoms, and even 12 patients did not recover completely during the follow‐up period.

In our systematic review, we found that the CNS manifestations did not appear to be correlated with the course and severity of peripheral neuropathy, which posed a challenge for making a clear diagnosis. Furthermore, clinicians may have a harder time making a correct diagnosis for patients with episodic CNS deficits as the first symptom. In these cases, CMTX1 should be distinguished from other episodic neurological diseases, including transient ischemic attack (TIA), mitochondrial encephalopathy with lactic acidosis and stroke‐like episodes (MELAS), acute disseminated encephalomyelitis (ADEM), and adrenoleukodystrophy (ALD).^71^, ^72^, ^73^, ^74^ At this time, it is important to perform a detailed family history assessment and neurological examination. If the patients have a positive family history or their neurological examinations reveal diminished deep tendon reflexes and pes cavus, these critical findings will provide supportive clues to a diagnosis of CMTX1. Besides, NCS could offer further help in the differential diagnosis. Most of the NCS results were abnormal in the present study, including prolonged latency, reduced amplitude, and slowed conduction velocity. Meanwhile, we found that the median MNCV was the most detectable and valuable parameter, ranging from 25 to 45 m/s in our cases. Additionally, more than two‐thirds of the patients underwent lumbar puncture. The results of CSF analysis were normal in most cases, while only a few had slightly elevated CSF protein levels or cell counts. Thus, it is difficult to use CSF examination as the main basis for distinguishing between CMTX1 and other episodic neurological diseases.

The final diagnosis of CMTX1 relies on the sequencing of the *GJB1* gene. To date, more than 400 *GJB1* mutations have been identified.^75^ Moreover, we found that 39 of these genetic mutations were related to episodic CNS dysfunction. These mutations usually occurred in the coding region, but in 2019, Luo et al. reported one mutation (−170T > G) located in the noncoding region, implying that clinicians should not only focus on the coding region to increase the positive detection rate.^14^ In addition, we observed the mutation c.425G > A (p.Arg142Gln) in three, c.65G > A (p.Arg22Gln) in two, and c.490C > T (p.Arg164Trp) in two unrelated patients, suggesting that CMTX1 patients carrying these three mutations might be more likely to develop CNS phenotype. Therefore, in the near future, these three candidate mutations can be expected to serve as genetic targets for knock‐in mice to explore the molecular aspects of the CNS phenotype. As for clinical treatment, there was no statistical difference in the course of disease with or without the use of corticosteroids and/or IVIg. Thus, we do not support the use of corticosteroids or IVIg to treat transient leukoencephalopathy caused by *GJB1* mutation.

## Conclusions

In summary, we have reported the most comprehensive summary of the demographic and clinical profile from 47 CMTX1 patients with episodic CNS dysfunction and provided new insight into the phenotype spectrum of CMTX1. In clinical practice, CMTX1 should be considered in patients presenting with episodic CNS dysfunction and abnormal white matter signals on MRI. Moreover, detailed neurological examination and NCS will provide critical clues to a correct diagnosis of CMTX1.

## Conflict of interest

The authors declare no financial or other conflict of interests.

## Supporting information


**Table S1.** Re‐analysis of the pathogenicity for all the mutations in CMTX1 patients with episodic neurological dysfunction

**Table S2.** The clinical manifestation in CMTX1 patients with episodic neurological dysfunction

**Table S3.** The results of MRI and CSF analysis in CMTX1 patients with episodic neurological dysfunction

**Table S4.** The NCS results in CMTX1 patients with episodic neurological dysfunction
