# Supplementary material for: Systematic review of CMTX1 patients with episodic neurological dysfunction
Source: Ann Clin Transl Neurol. 2020 Dec 12;8(1):213–23. doi: 10.1002/acn3.51271 (PMC7818278; doi:10.1002/acn3.51271)
Supplement: Supplementary file 1 — Table S1. Re‐analysis of the pathogenicity for all the mutations in CMTX1 patients with episodic neurological dysfunction [file ACN3-8-213-s001.docx]

Table S1. Re-analysis of the pathogenicity for all the mutations in CMTX1 patients with episodic neurological dysfunction

| Patient  Number | Nucleotide  Transition | Amino acid  Substitution | GnomAD allele frequency | REVEL  scores | Pathogenicity analysis based on ACMG criteria | Supporting  Reference |
| --- | --- | --- | --- | --- | --- | --- |
| 1 | c.381C>G | p.Ile127Met | 0 | 0.826 | P (PS1+PM2+PP1+PP2+PP3+PP4) | [1, 2] |
| 2 | c.391C>T | p.Arg107Trp | 4.6e-5 | 0.691 | P (PS1+PS3+PM2+PP1+PP2+PP3+PP4) | [3-11] |
| 3 | c.622G>A | p.Glu208Lys | 0 | 0.974 | P (PS1+PS3+PM2+PP1+PP2+PP3+PP4) | [7, 8, 12-30] |
| 4 | c.-170T>G | NCR^#^ | 0 | UA | LP (PM1+PM2+PP1+PP4) | [31, 32] |
| 5 | c.380T>C | p.Ile127Thr | 0 | 0.826 | LP (PM2+PM5+PP2+PP3+PP4) | [33] |
| 6 | c.227T>C | p.Leu76Pro | 0 | 0.983 | LP (PM1+PM2+PP2+PP3+PP4) | [34, 35] |
| 7 | c.425G>A | p.Arg142Gln | 0 | 0.91 | P (PS1+PM2+PM5+PP1+PP2+PP3+PP4) | [19, 34, 36-45] |
| 8 | c.227T>G | p.Leu76Arg | 0 | 0.988 | LP (PM1+PM2+PP2+PP3+PP4) | [35, 46, 47] |
| 9 | c.425G>A | p.Arg142Gln | 0 | 0.91 | P (PS1+PM2+PM5+PP1+PP2+PP3+PP4) | [19, 36-45, 48] |
| 10 | c.563C>T | p.Thr188Ile | 0 | 0.958 | LP (PM1+PM2+PP1+PP2+PP3+PP4) | [19, 48] |
| 11 | c.103G>C | p.Val35Leu | 0 | 0.851 | LP (PM2+PP1+PP2+PP3+PP4) | [48] |
| 12 | c.542T>C | p.Val181Ala | 0 | 0.967 | LP (PM2+PM5+PP1+PP2+PP3+PP4) | [19, 49, 50] |
| 13 | c.271G>A | p.Val91Met | 0 | 0.963 | P (PS1+PS3+PM2+PP1+PP2+PP3+PP4) | [14, 29, 51-59] |
| 14 | c.223C>T | p.Arg75Trp | 0 | 0.974 | P (PS1+PS3+PM2+PM5+PP2+PP3+PP4) | [6, 35, 60-67] |
| 15 | c.283G>A | p.Val95Met | 0 | 0.962 | P (PS1+PS3+PM2+PP1+PP2+PP3+PP4) | [5, 8, 14, 17, 22, 25, 44, 51, 54, 68-73] |
| 16 | c.445T>C | p.Phe149Leu | 0 | 0.971 | LP (PM2+PP1+PP2+PP3+PP4) | [4, 8, 22, 44, 51, 74-76] |
| 17 | c.467T>G | p.Leu156Arg | 0 | 0.982 | P (PS1+PS3+PM2+PM5+PP1+PP2+PP3+PP4) | [4, 8, 22, 44, 74, 75, 77] |
| 18 | c.490C>T | p.Arg164Trp | 5.82e-6 | 0.812 | P (PS1+PS3+PM2+PM5+PP1+PP2+PP3+PP4) | [8, 54, 58, 68, 78-87] |
| 19 | c.425G>A | p.Arg142Gln | 0 | 0.91 | P (PS1+PM2+PM5+PP1+PP2+PP3+PP4) | [19, 36-45, 88] |
| 20 | c.278T>G | p.Met93Arg | 0 | 0.968 | LP (PM2+PM5+PP2+PP3+PP4) | [89] |
| 21 | c.297_298  insCAA | p.Gln99_His  100insGln | 0 | UA | LP (PM2+PM4+PP2+PP4) | [90] |
| 22 | c.98T>A | p.lle33Asn | 0 | 0.888 | LP (PM1+PM2+PP2+PP3+PP4) | [62, 91] |
| 23 | c.3G>T | p.Met1Ile | 0 | 0.968 | P (PS3+PM2+PM5+PP1+PP2+PP3+PP4) | [92] |
| 24 | c.179G>A | p.Cys60Tyr | 0 | 0.947 | LP (PM2+PM5+PP1+PP2+PP3+PP4) | [12, 93-96] |
| 25 | c.260C>G | p.Pro87Leu | 0 | 0.969 | LP (PM2+PM5+PP2+PP3+PP4) | [97, 98] |
| 26 | c.477G>A | p.Val139Met | 5.84e-6 | 0.673 | P (PS1+PS3+PM2+PP1+PP2+PP3+PP4) | [6, 13, 44, 51, 53, 74, 93, 98, 99] |
| 27 | c.161A>G | p.Asn54Ser | 0 | 0.878 | LP (PM2+PP1+PP2+PP3+PP4) | [100] |
| 28 | c.396G>A | p.Trp132* | 0 | UA | P (PVS1+PS3+PM2+PP4) | [101, 102] |
| 29 | c.397delT | p.Trp133* | 0 | UA | P (PVS1+PS3+PM2+PP4) | [103] |
| 30 | c.196G>A | p.Asp66Asn | 0 | 0.864 | LP (PM1+PM2+PP2+PP3+PP4) | [8, 104] |
| 31 | c.556G>T | p.Glu186* | 0 | UA | P (PVS1+PS1+PS3+PM2+PP1+PP4) | [105, 106] |
| 32 | c.80T>C | p.Val27Ala | 0 | 0.965 | LP (PM1+PM2+PP2+PP3+PP4) | [107] |
| 33 | c.65G>A | p.Arg22Gln | 0 | 0.872 | P (PS1+PS2+PS3+PM2+PP1+PP2+PP3+PP4) | [6, 8, 9, 22, 27, 78, 79, 108-115] |
| 34 | c.491G>A | p.Arg164Gln | 5.8e-6 | 0.846 | P (PS1+PS3+PM2+PM5+PP2+PP3+PP4) | [8, 27, 44, 54, 57, 58, 78, 81, 85, 116-123] |
| 35 | c.530T>C | p.Val177Ala | 0 | 0.986 | LP (PM2+PM5+PP1+PP2+PP3+PP4) | [39, 124] |
| 36 | c.65G>A | p.Arg22Gln | 0 | 0.872 | P (PS1+PS2+PS3+PM2+PP1+PP2+PP3+PP4) | [6, 8, 9, 22, 27, 78, 79, 108-114, 125] |
| 37 | c.417G>A&  c.419C>G | p.Val139Met | 5.84e-6 | 0.978 | P (PS3+PM2+PP1+PP2+PP3+PP4) | [6, 13, 44, 51, 53, 74, 93, 99, 126] |
| 38 | c.417G>A&  c.419C>G | p.Val139Met | 5.84e-6 | 0.978 | P (PS3+PM2+PP1+PP2+PP3+PP4) | [6, 13, 44, 51, 53, 74, 93, 99, 126] |
| 39 | c.285C>T | p.Arg75Trp | 0 | 0.974 | P (PS1+PS3+PM2+PM5+PP1+PP2+PP3+PP4) | [6, 35, 60-66] |
| 40 | c.304_306  delGAG | p.102delGlu | 0 | UA | P (PS1+PM2+PM4+PP1+PP4) | [127] |
| 41 | c.304_306  delGAG | p.102delGlu | 0 | UA | P (PS1+PM2+PM4+PP1+PP4) | [127] |
| 42 | c.304_306  delGAG | p.102delGlu | 0 | UA | P (PS1+PM2+PM4+PP1+PP4) | [127] |
| 43 | c.490C>T | p.Arg164Trp | 5.82e-6 | 0.812 | P (PS1+PS3+PM2+PP1+PP2+PP3+PP4) | [8, 54, 58, 68, 78-86] |
| 44 | c.424C>T | p.Arg142Trp | 5.88e-6 | 0.915 | P (PS1+PS3+PM2+PP1+PP2+PP3+PP4) | [13, 19, 37, 54, 68, 74, 128-130] |
| 45 | c.565G>A | p.Cys168Tyr | 0 | 0.932 | LP (PS3+PM2+PP2+PP3+PP4) | [131] |
| 46 | c.164C>T | p.Ile55Thr | 0 | 0.944 | P (PS1+PS3+PM2+PM5+PP1+PP2+PP3+PP4) | [132, 133] |
| 47 | c.164C>T | p.Ile55Thr | 0 | 0.944 | P (PS1+PS3+PM2+PM5+PP1+PP2+PP3+PP4) | [132, 133] |

ACMG: American College of Medical Genetics; P: Pathogenic; PS: pathogenic strong; PM: pathogenic moderate; PP: pathogenic supporting; NCR: non-coding region; UA : unavailable; LP : Likely Pathogenic; PVS: pathogenic very strong

1. Nicholson, G.A., L. Yeung, and A. Corbett, *Efficient neurophysiologic selection of X-linked Charcot-Marie-Tooth families: ten novel mutations.* Neurology, 1998. **51**(5): p. 1412-6.

2. Tziakouri, A., et al., *Transient, Recurrent Central Nervous System Clinical Manifestations of X-Linked Charcot-Marie-Tooth Disease Presenting with Very Long Latency Periods between Episodes: Is Prolonged Sun Exposure a Provoking Factor?* Case Rep Neurol Med, 2020. **2020**: p. 9753139.

3. Tan, C.C., et al., *Novel mutations in the connexin 32 gene associated with X-linked Charcot-Marie tooth disease.* Hum Mutat, 1996. **7**(2): p. 167-71.

4. Latour, P., et al., *New mutations in the X-linked form of Charcot-Marie-Tooth disease.* Eur Neurol, 1997. **37**(1): p. 38-42.

5. Rouger, H., et al., *Charcot-Marie-Tooth disease with intermediate motor nerve conduction velocities: characterization of 14 Cx32 mutations in 35 families.* Hum Mutat, 1997. **10**(6): p. 443-52.

6. Silander, K., et al., *Screening for connexin 32 mutations in Charcot-Marie-Tooth disease families with possible X-linked inheritance.* Hum Genet, 1997. **100**(3-4): p. 391-7.

7. Mersiyanova, I.V., et al., *Screening for mutations in the peripheral myelin genes PMP22, MPZ and Cx32 (GJB1) in Russian Charcot-Marie-Tooth neuropathy patients.* Hum Mutat, 2000. **15**(4): p. 340-7.

8. Wang, H.L., et al., *Functional analysis of connexin-32 mutants associated with X-linked dominant Charcot-Marie-Tooth disease.* Neurobiol Dis, 2004. **15**(2): p. 361-70.

9. Miltenberger-Miltenyi, G., et al., *Identification and in silico analysis of 14 novel GJB1, MPZ and PMP22 gene mutations.* Eur J Hum Genet, 2009. **17**(9): p. 1154-9.

10. Milley, G.M., et al., *Three novel mutations and genetic epidemiology analysis of the Gap Junction Beta 1 (GJB1) gene among Hungarian Charcot-Marie-Tooth disease patients.* Neuromuscul Disord, 2016. **26**(10): p. 706-711.

11. Niu, J., et al., *GJB1 Mutation-A Disease Spectrum: Report of Case Series.* Frontiers in Neurology, 2020. **10**.

12. Fairweather, N., et al., *Mutations in the connexin 32 gene in X-linked dominant Charcot-Marie-Tooth disease (CMTX1).* Hum Mol Genet, 1994. **3**(1): p. 29-34.

13. Deschênes, S.M., et al., *Altered trafficking of mutant connexin32.* J Neurosci, 1997. **17**(23): p. 9077-84.

14. Ressot, C., et al., *Connexin32 mutations associated with X-linked Charcot-Marie-Tooth disease show two distinct behaviors: loss of function and altered gating properties.* J Neurosci, 1998. **18**(11): p. 4063-75.

15. Barrio, L.C., C. Castro, and J.M. Gómez-Hernandez, *Altered assembly of gap junction channels caused by COOH-terminal connexin32 mutants of CMTX.* Ann N Y Acad Sci, 1999. **883**: p. 526-9.

16. Castro, C., et al., *Altered formation of hemichannels and gap junction channels caused by C-terminal connexin-32 mutations.* J Neurosci, 1999. **19**(10): p. 3752-60.

17. Hahn, A.F., et al., *Genotype/phenotype correlations in X-linked dominant Charcot-Marie-Tooth disease.* Ann N Y Acad Sci, 1999. **883**: p. 366-82.

18. Martin, P.E., et al., *Analysis of gap junction assembly using mutated connexins detected in Charcot-Marie-Tooth X-linked disease.* J Neurochem, 2000. **74**(2): p. 711-20.

19. VanSlyke, J.K., S.M. Deschenes, and L.S. Musil, *Intracellular transport, assembly, and degradation of wild-type and disease-linked mutant gap junction proteins.* Mol Biol Cell, 2000. **11**(6): p. 1933-46.

20. Kochański, A. and D. Kabzińska, *Molecular genetic analysis of the GJB1 gene: a study of six mutations.* J Appl Genet, 2004. **45**(1): p. 95-100.

21. Song, S., et al., *Mutation frequency for Charcot-Marie-Tooth disease type 1 in the Chinese population is similar to that in the global ethnic patients.* Genetics in medicine : official journal of the American College of Medical Genetics, 2006. **8**(8): p. 532-535.

22. Shy, M.E., et al., *CMT1X phenotypes represent loss of GJB1 gene function.* Neurology, 2007. **68**(11): p. 849-55.

23. Song, S.J., et al., *[The same mutation Glu208Lys in the GJB1 gene was detected in 2 families with X-linked Charcot-Marie-Tooth disease].* Yi Chuan, 2007. **29**(7): p. 800-4.

24. Siskind, C.E., et al., *Phenotype expression in women with CMT1X.* J Peripher Nerv Syst, 2011. **16**(2): p. 102-7.

25. Kim, Y., et al., *X-linked dominant Charcot-Marie-Tooth disease with connexin 32 (Cx32) mutations in Koreans.* Clin Genet, 2012. **81**(2): p. 142-9.

26. Manganelli, F., et al., *Charcot-Marie-Tooth disease: frequency of genetic subtypes in a Southern Italy population.* J Peripher Nerv Syst, 2014. **19**(4): p. 292-8.

27. Wang, R., et al., *Clinical and genetic spectra in a series of Chinese patients with Charcot-Marie-Tooth disease.* Clin Chim Acta, 2015. **451**(Pt B): p. 263-70.

28. Laššuthová, P., et al., *Improving diagnosis of inherited peripheral neuropathies through gene panel analysis.* Orphanet J Rare Dis, 2016. **11**(1): p. 118.

29. Hong, Y.B., et al., *Clinical characterization and genetic analysis of Korean patients with X-linked Charcot-Marie-Tooth disease type 1.* J Peripher Nerv Syst, 2017. **22**(3): p. 172-181.

30. Li, Q., et al., *Recurrent Stroke-Like Symptoms After Cesarean Section Deliveries in a Female Patient With X-Linked Charcot-Marie-Tooth Type 1.* Frontiers in Neurology, 2020. **11**.

31. Luo, S., et al., *A Novel Variant in Non-coding Region of GJB1 Is Associated With X-Linked Charcot-Marie-Tooth Disease Type 1 and Transient CNS Symptoms.* Front Neurol, 2019. **10**: p. 413.

32. Houlden, H., et al., *Connexin 32 promoter P2 mutations: a mechanism of peripheral nerve dysfunction.* Ann Neurol, 2004. **56**(5): p. 730-4.

33. Hu, G., et al., *Novel gap junction protein beta-1 gene mutation associated with a stroke-like syndrome and central nervous system involvement in patients with X-linked Charcot-Marie-Tooth Type 1: A case report and literature review.* Clin Neurol Neurosurg, 2019. **180**: p. 68-73.

34. Hardy, D.I., et al., *X-linked Charcot-Marie-Tooth Disease Presenting with Stuttering Stroke-like Symptoms.* Neuropediatrics, 2019. **50**(5): p. 304-307.

35. Abrams, C.K., et al., *Functional requirement for a highly conserved charged residue at position 75 in the gap junction protein connexin 32.* J Biol Chem, 2013. **288**(5): p. 3609-19.

36. Bergoffen, J., et al., *Connexin mutations in X-linked Charcot-Marie-Tooth disease.* Science (New York, N.Y.), 1993. **262**(5142): p. 2039-2042.

37. Ionasescu, V., C. Searby, and R. Ionasescu, *Point mutations of the connexin32 (GJB1) gene in X-linked dominant Charcot-Marie-Tooth neuropathy.* Hum Mol Genet, 1994. **3**(2): p. 355-8.

38. Bone, L.J., et al., *Connexin32 and X-linked Charcot-Marie-Tooth disease.* Neurobiol Dis, 1997. **4**(3-4): p. 221-30.

39. Ikegami, T., et al., *Four novel mutations of the connexin 32 gene in four Japanese families with Charcot-Marie-Tooth disease type 1.* American journal of medical genetics, 1998. **80**(4): p. 352-355.

40. Scherer, S.S., et al., *The role of the gap junction protein connexin32 in the pathogenesis of X-linked Charcot-Marie-Tooth disease.* Novartis Found Symp, 1999. **219**: p. 175-85; discussion 185-7.

41. Stojkovic, T., et al., *Sensorineural deafness in X-linked Charcot-Marie-Tooth disease with connexin 32 mutation (R142Q).* Neurology, 1999. **52**(5): p. 1010-4.

42. Dubourg, O., et al., *Clinical, electrophysiological and molecular genetic characteristics of 93 patients with X-linked Charcot-Marie-Tooth disease.* Brain : a journal of neurology, 2001. **124**(Pt 10): p. 1958-1967.

43. Lorefice, L., et al., *Charcot-Marie-Tooth disease: genetic subtypes in the Sardinian population.* Neurological sciences : official journal of the Italian Neurological Society and of the Italian Society of Clinical Neurophysiology, 2017. **38**(6): p. 1019-1025.

44. Nykamp, K., et al., *Sherloc: a comprehensive refinement of the ACMG-AMP variant classification criteria.* Genet Med, 2017. **19**(10): p. 1105-1117.

45. Yuan, J.H., et al., *Genetic and phenotypic profile of 112 patients with X-linked Charcot-Marie-Tooth disease type 1.* European journal of neurology, 2018. **25**(12): p. 1454-1461.

46. Miki, Y., et al., *A family with IVIg-responsive Charcot-Marie-Tooth disease.* Journal of neurology, 2013. **260**(4): p. 1147-1151.

47. Santoro, J.D. and T. Chitnis, *Strokelike Episodes in a Patient With Chronic Gait Abnormalities.* JAMA Neurol, 2019. **76**(5): p. 621-622.

48. Liang, Y., et al., *Recurrent episodes of reversible posterior leukoencephalopathy in three Chinese families with GJB1 mutations in X-linked Charcot-Marie-tooth type 1 disease: cases report.* BMC Neurol, 2019. **19**(1): p. 325.

49. Abrams, C.K., et al., *Pathogenesis of X-linked Charcot-Marie-Tooth disease: differential effects of two mutations in connexin 32.* The Journal of neuroscience : the official journal of the Society for Neuroscience, 2003. **23**(33): p. 10548-10558.

50. Aktan, Z., et al., *A case with CMTX1 disease showing transient ischemic-attack-like episodes.* Neurol Neurochir Pol, 2018. **52**(2): p. 285-288.

51. Bone, L.J., et al., *New connexin32 mutations associated with X-linked Charcot-Marie-Tooth disease.* Neurology, 1995. **45**(10): p. 1863-6.

52. Bissar-Tadmouri, N., et al., *Mutational analysis and genotype/phenotype correlation in Turkish Charcot-Marie-Tooth Type 1 and HNPP patients.* Clin Genet, 2000. **58**(5): p. 396-402.

53. Abrams, C.K., et al., *Functional alterations in gap junction channels formed by mutant forms of connexin 32: evidence for loss of function as a pathogenic mechanism in the X-linked form of Charcot-Marie-Tooth disease.* Brain Res, 2001. **900**(1): p. 9-25.

54. Dubourg, O., et al., *Clinical, electrophysiological and molecular genetic characteristics of 93 patients with X-linked Charcot-Marie-Tooth disease.* Brain, 2001. **124**(Pt 10): p. 1958-67.

55. Michell, A.W., et al., *GJB1 gene mutations in suspected inflammatory demyelinating neuropathies not responding to treatment.* J Neurol Neurosurg Psychiatry, 2009. **80**(6): p. 699-700.

56. Arthur-Farraj, P.J., et al., *Hand weakness in Charcot-Marie-Tooth disease 1X.* Neuromuscul Disord, 2012. **22**(7): p. 622-6.

57. Li, L.X., et al., *Improving molecular diagnosis of Chinese patients with Charcot-Marie-Tooth by targeted next-generation sequencing and functional analysis.* Oncotarget, 2016. **7**(19): p. 27655-64.

58. Tsai, P.C., et al., *Clinical and biophysical characterization of 19 GJB1 mutations.* Ann Clin Transl Neurol, 2016. **3**(11): p. 854-865.

59. Nicholson, P.D. and S.M. Pulst, *Centrally involved X-linked Charcot-Marie-Tooth disease presenting as a stroke-mimic.* Neurol Genet, 2017. **3**(1): p. e128.

60. Latour, P., et al., *Mutations in the X-linked form of Charcot-Marie-Tooth disease in the French population.* Neurogenetics, 1997. **1**(2): p. 117-23.

61. Numakura, C., et al., *Molecular analysis in Japanese patients with Charcot-Marie-Tooth disease: DGGE analysis for PMP22, MPZ, and Cx32/GJB1 mutations.* Hum Mutat, 2002. **20**(5): p. 392-8.

62. Yum, S.W., et al., *Diverse trafficking abnormalities of connexin32 mutants causing CMTX.* Neurobiol Dis, 2002. **11**(1): p. 43-52.

63. Taylor, R.A., et al., *The CNS phenotype of X-linked Charcot-Marie-Tooth disease: more than a peripheral problem.* Neurology, 2003. **61**(11): p. 1475-8.

64. Zhang, R.X., et al., *Mutation screening of Cx32 in Han Chinese patients with Charcot-Marie-Tooth disease.* Beijing Da Xue Xue Bao Yi Xue Ban, 2005. **37**(1): p. 68-71.

65. Casasnovas, C., et al., *Clinical and molecular analysis of X-linked Charcot-Marie-Tooth disease type 1 in Spanish population.* Clin Genet, 2006. **70**(6): p. 516-23.

66. Sargiannidou, I., et al., *Connexin32 mutations cause loss of function in Schwann cells and oligodendrocytes leading to PNS and CNS myelination defects.* J Neurosci, 2009. **29**(15): p. 4736-49.

67. Parissis, D., et al., *Charcot-Marie-Tooth Disease 1X Simulating Paraparetic Guillain-Barre Syndrome.* Neurologist, 2017. **22**(6): p. 234-236.

68. Ionasescu, V.V., *X-linked Charcot-Marie-Tooth disease and connexin32.* Cell Biol Int, 1998. **22**(11-12): p. 807-13.

69. Park, H.K., et al., *Mutation analysis of the PMP22, MPZ, EGR2, LITAF, and GJB1 genes in Korean patients with Charcot-Marie-Tooth neuropathy type 1.* Clin Genet, 2006. **70**(3): p. 253-6.

70. Baaj, Y., et al., *A highly specific microarray method for point mutation detection.* Biotechniques, 2008. **44**(1): p. 119-26.

71. Baaj, Y., et al., *Multiplex detection and genotyping of point mutations involved in charcot-marie-tooth disease using a hairpin microarray-based assay.* Res Lett Biochem, 2009. **2009**: p. 960560.

72. Montenegro, G., et al., *Exome sequencing allows for rapid gene identification in a Charcot-Marie-Tooth family.* Ann Neurol, 2011. **69**(3): p. 464-70.

73. Kim, J.K., S.A. Han, and S.J. Kim, *X-linked Charcot-Marie-Tooth disease with GJB1 mutation presenting as acute disseminated encephalomyelitis-like illness: A case report.* Medicine (Baltimore), 2017. **96**(49): p. e9176.

74. Bergoffen, J., et al., *Connexin mutations in X-linked Charcot-Marie-Tooth disease.* Science, 1993. **262**(5142): p. 2039-42.

75. Wu, N., et al., *Recurrent Episodes of Stroke-Like Symptoms in a Patient with Charcot-Marie-Tooth Neuropathy X Type 1.* Case Rep Neurol, 2015. **7**(3): p. 247-52.

76. Xie, C., et al., *CNS involvement in CMTX1 caused by a novel connexin 32 mutation: a 6-year follow-up in neuroimaging and nerve conduction.* Neurol Sci, 2016. **37**(7): p. 1063-70.

77. Bone, L.J., et al., *New connexin32 mutations associated with X-linked Charcot-Marie-Tooth disease.* Neurology, 1995. **45**(10): p. 1863-1866.

78. Ionasescu, V., et al., *New point mutations and deletions of the connexin 32 gene in X-linked Charcot-Marie-Tooth neuropathy.* Neuromuscul Disord, 1995. **5**(4): p. 297-9.

79. Ionasescu, V., R. Ionasescu, and C. Searby, *Correlation between connexin 32 gene mutations and clinical phenotype in X-linked dominant Charcot-Marie-Tooth neuropathy.* Am J Med Genet, 1996. **63**(3): p. 486-91.

80. Oterino, A., et al., *Arginine-164-tryptophan substitution in connexin32 associated with X linked dominant Charcot-Marie-Tooth disease.* J Med Genet, 1996. **33**(5): p. 413-5.

81. Bort, S., et al., *Mutational analysis of the MPZ, PMP22 and Cx32 genes in patients of Spanish ancestry with Charcot-Marie-Tooth disease and hereditary neuropathy with liability to pressure palsies.* Hum Genet, 1997. **99**(6): p. 746-54.

82. Young, P., et al., *Mutation analysis in Chariot-Marie Tooth disease type 1: point mutations in the MPZ gene and the GJB1 gene cause comparable phenotypic heterogeneity.* J Neurol, 2001. **248**(5): p. 410-5.

83. Schelhaas, H.J., et al., *Transient cerebral white matter lesions in a patient with connexin 32 missense mutation.* Neurology, 2002. **59**(12): p. 2007-8.

84. Isoardo, G., et al., *X-linked Charcot-Marie-Tooth disease and progressive-relapsing central demyelinating disease.* Neurology, 2005. **65**(10): p. 1672-3.

85. Høyer, H., et al., *Genetic diagnosis of Charcot-Marie-Tooth disease in a population by next-generation sequencing.* Biomed Res Int, 2014. **2014**: p. 210401.

86. Lu, Y.Y., et al., *Clinical and Genetic Features of Chinese X-linked Charcot-Marie-Tooth Type 1 Disease.* Chin Med J (Engl), 2017. **130**(9): p. 1049-1054.

87. Shu, X.M., et al., *X-Linked Hereditary Motor Sensory Neuropathy Type 1 (CMTX1) in a Three-Generation Gelao Chinese Family.* Neuropediatrics, 2015. **46**(6): p. 424-7.

88. Kulkarni, G.B., et al., *Episodic neurological dysfunction in hereditary peripheral neuropathy.* Ann Indian Acad Neurol, 2015. **18**(1): p. 111-4.

89. Zhao, Y., et al., *Transient, recurrent, white matter lesions in x-linked Charcot-Marie-tooth disease with novel mutation of gap junction protein beta 1 gene in China: a case report.* BMC neurology, 2014. **14**: p. 156.

90. Sagnelli, A., et al., *X-linked Charcot-Marie-Tooth type 1: stroke-like presentation of a novel GJB1 mutation.* J Peripher Nerv Syst, 2014. **19**(2): p. 183-6.

91. McKinney, J.L., et al., *Recurrent central nervous system white matter changes in charcot-Marie-tooth type X disease.* Muscle & nerve, 2014. **49**(3): p. 451-454.

92. Kim, G.-H., et al., *Charcot-Marie-Tooth disease masquerading as acute demyelinating encephalomyelitis-like illness.* Pediatrics, 2014. **134**(1): p. e270-e273.

93. Omori, Y., M. Mesnil, and H. Yamasaki, *Connexin 32 mutations from X-linked Charcot-Marie-Tooth disease patients: functional defects and dominant negative effects.* Mol Biol Cell, 1996. **7**(6): p. 907-16.

94. *3rd workshop of the European CMT consortium: 54th ENMC International Workshop on genotype/phenotype correlations in Charcot-Marie-Tooth type 1 and hereditary neuropathy with liability to pressure palsies 28-30 November 1997, Naarden, The Netherlands.* Neuromuscul Disord, 1998. **8**(8): p. 591-603.

95. Sorour, E. and M. Upadhyaya, *Mutation analysis in Charcot-Marie-Tooth disease type 1 (CMT1).* Hum Mutat, 1998. **Suppl 1**: p. S242-7.

96. Appu, M. and S. Mar, *Novel familial pathogenic mutation in gap junction protein, beta-1 gene (GJB1) associated with transient neurological deficits in a patient with X-linked Charcot-Marie-Tooth disease.* Muscle Nerve, 2014. **50**(6): p. 1023-4.

97. Kuntzer, T., et al., *Phenotypic expression of a Pro 87 to Leu mutation in the connexin 32 gene in a large Swiss family with Charcot-Marie-Tooth neuropathy.* Journal of the neurological sciences, 2003. **207**(1-2): p. 77-86.

98. Al-Mateen, M., A.K. Craig, and P.F. Chance, *The central nervous system phenotype of X-linked Charcot-Marie-Tooth disease: a transient disorder of children and young adults.* J Child Neurol, 2014. **29**(3): p. 342-8.

99. Numakura, C., et al., *Molecular analysis in Japanese patients with Charcot-Marie-Tooth disease: DGGE analysis for PMP22, MPZ, and Cx32/GJB1 mutations.* Human mutation, 2002. **20**(5): p. 392-398.

100. Zhong, L., et al., *Clinical reasoning: a young man with reversible paralysis, cerebral white matter lesions, and peripheral neuropathy.* Neurology, 2012. **79**(8): p. e70-2.

101. Lin, C., et al., *Deletion and nonsense mutations of the connexin 32 gene associated with Charcot-Marie-Tooth disease.* The Tohoku journal of experimental medicine, 1999. **188**(3): p. 239-244.

102. Sato, K., et al., *Diffusion tensor imaging and magnetic resonance spectroscopy of transient cerebral white matter lesions in X-linked Charcot-Marie-Tooth disease.* J Neurol Sci, 2012. **316**(1-2): p. 178-80.

103. Sakaguchi, H., et al., *A novel GJB1 frameshift mutation produces a transient CNS symptom of X-linked Charcot-Marie-Tooth disease.* Journal of neurology, 2011. **258**(2): p. 284-290.

104. U-King-Im, J.M., et al., *MRI findings in X-linked Charcot-Marie-Tooth disease associated with a novel connexin 32 mutation.* Clinical radiology, 2011. **66**(5): p. 471-474.

105. Ionasescu, V., et al., *New point mutations and deletions of the connexin 32 gene in X-linked Charcot-Marie-Tooth neuropathy.* Neuromuscular disorders : NMD, 1995. **5**(4): p. 297-299.

106. Basu, A., et al., *Recurrent stroke-like episodes in X-linked Charcot-Marie-Tooth disease.* Neurology, 2011. **77**(12): p. 1205-6.

107. Absoud, M., et al., *Hereditary motor sensory neuropathy (type 1) presenting with transient and persistent central nervous system manifestations: a novel genetic mutation.* Developmental medicine and child neurology, 2011. **53**(4): p. 381-382.

108. Nelis, E., et al., *Estimation of the mutation frequencies in Charcot-Marie-Tooth disease type 1 and hereditary neuropathy with liability to pressure palsies: a European collaborative study.* Eur J Hum Genet, 1996. **4**(1): p. 25-33.

109. Senderek, J., et al., *X-linked dominant Charcot-Marie-Tooth disease: nerve biopsies allow morphological evaluation and detection of connexin32 mutations (Arg15Trp, Arg22Gln).* Acta Neuropathol, 1998. **95**(5): p. 443-9.

110. Silander, K., et al., *Spectrum of mutations in Finnish patients with Charcot-Marie-Tooth disease and related neuropathies.* Hum Mutat, 1998. **12**(1): p. 59-68.

111. Senderek, J., et al., *X-linked dominant Charcot-Marie-Tooth neuropathy: clinical, electrophysiological, and morphological phenotype in four families with different connexin32 mutations(1).* J Neurol Sci, 1999. **167**(2): p. 90-101.

112. Matsuyama, W., et al., *Phenotypes of X-linked Charcot-Marie-Tooth disease and altered trafficking of mutant connexin 32 (GJB1).* J Hum Genet, 2001. **46**(6): p. 307-13.

113. Boerkoel, C.F., et al., *Charcot-Marie-Tooth disease and related neuropathies: mutation distribution and genotype-phenotype correlation.* Ann Neurol, 2002. **51**(2): p. 190-201.

114. Takashima, H., et al., *Gap junction protein beta 1 (GJB1) mutations and central nervous system symptoms in X-linked Charcot-Marie-Tooth disease.* Acta neurologica Scandinavica, 2003. **107**(1): p. 31-37.

115. Rosser, T., et al., *Transient leukoencephalopathy associated with X-linked Charcot-Marie-Tooth disease.* J Child Neurol, 2010. **25**(8): p. 1013-6.

116. Yoshihara, T., et al., *Mutations in the peripheral myelin protein zero and connexin32 genes detected by non-isotopic RNase cleavage assay and their phenotypes in Japanese patients with Charcot-Marie-Tooth disease.* Hum Mutat, 2000. **16**(2): p. 177-8.

117. Huehne, K., et al., *Novel mutations in the Charcot-Marie-Tooth disease genes PMP22, MPZ, and GJB1.* Hum Mutat, 2003. **21**(1): p. 100.

118. Choi, B.O., et al., *Mutational analysis of PMP22, MPZ, GJB1, EGR2 and NEFL in Korean Charcot-Marie-Tooth neuropathy patients.* Hum Mutat, 2004. **24**(2): p. 185-6.

119. Casasnovas, C., et al., *Clinical and molecular analysis of X-linked Charcot-Marie-Tooth disease type 1 in Spanish population.* Clinical genetics, 2006. **70**(6): p. 516-523.

120. Karadima, G., et al., *Mutational analysis of PMP22, GJB1 and MPZ in Greek Charcot-Marie-Tooth type 1 neuropathy patients.* Clin Genet, 2011. **80**(5): p. 497-9.

121. Braathen, G.J., *Genetic epidemiology of Charcot-Marie-Tooth disease.* Acta Neurol Scand Suppl, 2012(193): p. iv-22.

122. Nam, S.H., et al., *Identification of Genetic Causes of Inherited Peripheral Neuropathies by Targeted Gene Panel Sequencing.* Mol Cells, 2016. **39**(5): p. 382-8.

123. Fusco, C., et al., *Coexistent central and peripheral nervous system involvement in a Charcot-Marie-Tooth syndrome X-linked patient.* J Child Neurol, 2010. **25**(6): p. 759-63.

124. Anand, G., et al., *X-linked hereditary motor sensory neuropathy (type 1) presenting with a stroke-like episode.* Dev Med Child Neurol, 2010. **52**(7): p. 677-9.

125. Srinivasan, J., et al., *Central nervous system signs in X-linked Charcot-Marie-Tooth disease after hyperventilation.* Pediatr Neurol, 2008. **38**(4): p. 293-5.

126. Halbrich, M., et al., *A V139M mutation also causes the reversible CNS phenotype in CMTX.* Can J Neurol Sci, 2008. **35**(3): p. 372-4.

127. Hanemann, C.O., et al., *Transient, recurrent, white matter lesions in X-linked Charcot-Marie-Tooth disease with novel connexin 32 mutation.* Arch Neurol, 2003. **60**(4): p. 605-9.

128. Bruzzone, R., et al., *Null mutations of connexin32 in patients with X-linked Charcot-Marie-Tooth disease.* Neuron, 1994. **13**(5): p. 1253-60.

129. Mones, S., et al., *CMTX1 patients' cells present genomic instability corrected by CamKII inhibitors.* Orphanet J Rare Dis, 2015. **10**: p. 56.

130. Richards, S., et al., *Standards and guidelines for the interpretation of sequence variants: a joint consensus recommendation of the American College of Medical Genetics and Genomics and the Association for Molecular Pathology.* Genetics in medicine : official journal of the American College of Medical Genetics, 2015. **17**(5): p. 405-424.

131. Paulson, H.L., et al., *Transient central nervous system white matter abnormality in X-linked Charcot-Marie-Tooth disease.* Annals of neurology, 2002. **52**(4): p. 429-434.

132. Karadimas, C., et al., *Three novel mutations in the gap junction beta 1 (GJB1) gene coding region identified in Charcot-Marie-Tooth patients of Greek origin: T55I, R164Q, V120E. Mutation in brief no 236. Online.* Human mutation, 1999. **13**(4): p. 339.

133. Panas, M., et al., *Episodes of generalized weakness in two sibs with the C164T mutation of the connexin 32 gene.* Neurology, 2001. **57**(10): p. 1906-1908.
