# Supplementary material for: Systematic review of CMTX1 patients with episodic neurological dysfunction
Source: Ann Clin Transl Neurol. 2020 Dec 12;8(1):213–23. doi: 10.1002/acn3.51271 (PMC7818278; doi:10.1002/acn3.51271)
Supplement: Supplementary file 2 — Table S2. The clinical manifestation in CMTX1 patients with episodic neurological dysfunction [file ACN3-8-213-s002.docx]

Table S2. The clinical manifestations in CMTX1 with episodic neurological dysfunction

| Patient  Number | Central Nervous System Manifestations | Time to recovery | Episode(s) | Predisposing factor(s) | Peripheral Nervous System Manifestations |
| --- | --- | --- | --- | --- | --- |
| 1 | Right lower limbs weakness, dysarthria | 5h | Recurrent | Exposure to strong sunshine | Weakness in lower limbs |
| 2 | All limbs weakness, dysphasia, bilateral pyramidal signs | 3min | Recurrent | None | Normal |
| 3 | Right hemiplegia, right lingual weakness, dysarthria, bilateral pyramidal signs | 1m | Recurrent | Puerperium | Areflexia in limbs, diminished sense, pes cavus |
| 4 | Bilateral facial and all limbs weakness, dysarthria, ataxia, bilateral pyramidal signs | 6h | Recurrent | Thyroid  malfunction | Areflexia in limbs, pes cavus |
| 5 | Distal lower limbs weakness, dysarthria, dysphagia, lips and right upper limb numbness | 4.5h | Recurrent | None | Weakness in lower limbs, areflexia in all limbs, pes cavus |
| 6 | Right upper limb weakness and numbness, right facial weakness, dysarthria | 2d | Recurrent | Travel to high altitude | Atrophy in distal lower limbs, hyporeflexia in ankle, pes cavus |
| 7 | Right facial and lower limb weakness | 2d | Recurrent | Travel to high altitude | Areflexia in lower limb, diminished vibration sense in lower limbs, pes cavus |
| 8 | Left hemiplegia, dysarthria | 1h | Recurrent | None | Areflexia, diminished proprioceptive sense in lower limbs, pes cavus |
| 9 | All limbs weakness, dysarthria, chorea-choreiform movements, confusion | 3m | Recurrent | None | Atrophy of distal lower limbs, areflexia in lower limbs, pes cavus |
| 10 | All limbs weakness and numbness, dysarthria | Several hours | Recurrent | None | Hyporeflexia in all limbs, pes cavus |
| 11 | All limbs weakness and numbness, dysarthria, dysphagia, horizontal nystagmus on both eyes, ataxia, dizziness | 20h | Recurrent | Trauma | Atrophy in distal limbs, areflexia in all limbs, pes cavus |
| 12 | Hemiplegia, dysarthria, upgaze paresis, ataxia | 12h | Recurrent | None | Hyporeflexia, pes cavus, hammer toes |
| 13 | Dysarthria | Several hours | Recurrent | None | Areflexia in distal lower limbs , pes cavus |
| 14 | Distal lower limbs weakness | 2d | Single | Infection, poor sleep | Atrophy and absent sense in distal limbs, areflexia, diminished proprioceptive sense, pes cavus |
| 15 | Left hemiparesis, dysarthria | 9w | Recurrent | None | Atrophy in distal lower limbs, pes cavus |
| 16 | Hemiplegia, facial numbness, dysarthria | Less than 4h | Recurrent | Intensive exercise | Atrophy in all distal limbs, areflexia in distal lower limbs, biceps hyporeflexia |
| 17 | Limbs, left facial and lingual weakness, left hand numbness, dysarthria, ataxia | 6m | Recurrent | Poor sleep | Areflexia in lower limbs, hyporeflexia in upper limbs, pes cavus, hammer toes |
| 18 | Right hemiplegia, numbness, dysarthria, dysphasia, bilateral pyramidal signs | 36h | Recurrent | None | Areflexia in lower limbs, hyporeflexia in upper limbs, pes cavus |
| 19 | Right hemiparesis, right face numbness, dysarthria | 10h | Recurrent | None | Atrophy in distal limbs, areflexia in distal lower limbs and hyporeflexia in upper limbs, diminished vibration sense in all limbs, pes cavus, hammer toes |
| 20 | Limbs weakness, right limbs numbness, dysphagia, bilateral pyramidal signs | 3h | Recurrent | Infection | Atrophy in distal limbs, hyporeflexia in all limbs |
| 21 | Right hemiparesis, aphasia, abnormal sense in right limbs | Several hours | Recurrent | Travel to high altitude | Atrophy and weakness in distal lower limbs, absent proprioceptive sense, pes cavus |
| 22 | Left arm and right leg weakness, left facial numbness, dysphasia, dysarthria | 3d | Recurrent | Infection | Atrophy in lower limbs, hyporeflexia except at the right knee, diminished vibration sense distally, stocking-glove distribution of sensory loss, pes cavus, hammer toes |
| 23 | Hemiparesis, dysarthria, aphasia | 4d | Recurrent | None | Areflexia in ankle |
| 24 | Left hemiparesis, numbness, dysarthria, left pyramidal sign | 48h | Recurrent | None | Areflexia, diminished vibration sense in toes, pes cavus |
| 25 | Right hemiparesis, right hand numbness, right pyramidal sign | 3d | Recurrent | Travel to high altitude | Hyporeflexia, pes cavus, hammer toes |
| 26 | Quadriparesis, dysarthria, dysphagia | 12h | Recurrent | Fever | Atrophy in distal lower limbs, areflexia in all limbs, pes cavus |
| 27 | Quadriparesis, dysarthria, diplopia | 1d | Recurrent | Fever | Normal |
| 28 | Right hemiparesis, left arm weakness, dysarthria, lethargy, dilated pupils, loss of light reflex | 2d | Recurrent | None | Areflexia in all limbs, pes cavus |
| 29 | Left limbs weakness, left face and arm numbness, dysphagia | Several hours | Single | None | Atrophy and weakness in distal lower limbs, areflexia in lower limbs and hyporeflexia in upper limbs, pes cavus |
| 30 | Right upper limb weakness, aphasia, dizziness | 2.5h | Single | Infection | Atrophy in the hand |
| 31 | Hemiparesis, dysarthria | 1w | Recurrent | Intensive exercise | Hyporeflexia in ankle, diminished sense distally, pes cavus |
| 32 | Dysphagia, dysarthria, ataxia | UA | Recurrent | Infection | Atrophy in the first dorsal interossei, areflexia in all limbs, pes cavus |
| 33 | All limbs weakness, right arm numbness, bilateral pyramidal sign | Several weeks | Recurrent | None | Hyporeflexia |
| 34 | Distal lower limbs weakness, dysarthria, paralysis of the V, VII, and XII left cranial nerves, ataxia, disorientation for time and place | 1d | Single | Infection | Atrophy in distal limbs, hyporeflexia in ankle, pes cavus |
| 35 | Left hemiparesis, dysarthria | 36h | Single | Infection | Weakness in lower limbs, areflexia in ankles |
| 36 | Limbs hemiplegia, dysarthria, ataxia | 80min | Recurrent | Hyperventilation | Areflexia in ankles, absent sense in lower distal limbs |
| 37 | Left limbs weakness, dysarthria, bilateral pyramidal signs | 8h | Recurrent | Trauma | Areflexia |
| 38 | Right hemiplegia, dysarthria, dysphagia, ataxia | 31h | Single | Infection | Areflexia, diminished in temperature and vibration sense |
| 39 | Hemiparesis, right face and arm numbness, dysarthria, dysphagia, motor aphasia | Several hours | Recurrent | Intensive exercise | Hyporeflexia in ankle, pes cavus |
| 40 | Hemiplegia, pyramidal signs | 2d | Recurrent | Infection | Diminished vibration sense |
| 41 | Limbs weakness, dysarthria, motor aphasia, pyramidal signs | 3h | Recurrent | Intensive exercise | Diminished vibration sense, pes cavus |
| 42 | Bilateral facial weakness, dysarthria, dysphagia, paralysis of cranial nerves, pyramidal signs | 1h | Recurrent | Infection | Diminished vibration sense |
| 43 | Both facial and feet weakness, dysarthria, dysphagia, bilateral pyramidal signs | 6h | Single | Fever | Weakness in distal lower limbs, atrophy in distal limbs, areflexia in knee and ankle, diminished vibration sense in distal lower limbs, pes cavus, hammer toes |
| 44 | Dysarthria, ataxia | 2w | Single | Travel to high altitude | Atrophy and weakness in all distal limbs, areflexia |
| 45 | Limbs and lingual weakness, dysarthria, ataxia | 10h | Recurrent | Travel to high altitude | Weakness in all distal limbs, areflexia, diminished sense in distal limbs |
| 46 | Limbs weakness, dysarthria, dysphagia, dyspnea, lethargy, bilateral pyramidal signs | 3d | Recurrent | None | Atrophy and weakness in all distal limbs, areflexia, diminished sense in distal limbs, pes cavus |
| 47 | Limbs weakness | UA | Recurrent | None | Atrophy and weakness in all distal limbs, areflexia, diminished sense in distal limbs, pes cavus |

h: hour; min: minute; m: month; d: day; UA: Unavailable
