# Supplementary material for: Systematic review of CMTX1 patients with episodic neurological dysfunction
Source: Ann Clin Transl Neurol. 2020 Dec 12;8(1):213–23. doi: 10.1002/acn3.51271 (PMC7818278; doi:10.1002/acn3.51271)
Supplement: Supplementary file 3 — Table S3. The results of MRI and CSF analysis in CMTX1 patients with episodic neurological dysfunction [file ACN3-8-213-s003.docx]

Table S3. The results of MRI and CSF analysis in CMTX1 with episodic neurological dysfunction

| Patient  Number | MRI lesion | | | MRI sequence | | MRI recovery | CSF |
| --- | --- | --- | --- | --- | --- | --- | --- |
|  | Bilaterally white matter | Corpus callosum | Others | T2/FLAIR | DWI |  |  |
| 1 | Yes | Yes (splenium) | Frontal and parietal | Hyperintensity | Restricted | 1m | Normal |
| 2 | Yes | Normal | Normal | Hyperintensity | Restricted | 1m | Normal |
| 3 | Yes | Yes | Bilateral posterior limbs of internal capsule | Hyperintensity | Restricted | UA | UA |
| 4 | Yes | Yes (splenium) | Normal | Hyperintensity | Restricted | 5m | UA |
| 5 | Yes | Yes (splenium) | Normal | Hyperintensity | UA | 9d | Elevated protein levels (65.1 mg/dl) |
| 6 | Yes | Yes (splenium) | Left precentral gyrus | Hyperintensity | Restricted | UA | Normal |
| 7 | Yes | Yes (splenium) | Normal | Hyperintensity | Restricted | 4m | Elevated protein levels (139 mg/dl) |
| 8 | Yes | Yes | Normal | Hyperintensity | Restricted | UA | Normal |
| 9 | Yes | Yes (splenium) | Normal | UA | Restricted | 2w | Normal |
| 10 | Yes | Normal | Bilateral parieto-occipital regions | UA | Restricted | 18d | Normal |
| 11 | Yes | Yes (splenium) | Normal | UA | Restricted | 2w | UA |
| 12 | Yes | Yes | Normal | Hyperintensity | UA | 2w | Elevated protein levels (77.3 mg/dl) |
| 13 | Yes | Yes (splenium) | Normal | Hyperintensity | UA | 6m | UA |
| 15 | Yes | Yes (splenium) | Bilateral posterior limbs of internal capsule | UA | Restricted | 4m | Normal |
| 16 | Yes | Yes (splenium) | Bilateral parietal lobe | Hyperintensity | Restricted | Several months | UA |
| 17 | Yes | Yes (splenium) | Bilateral corticospinal tracts and middle cerebellar peduncles, parietal lobe | Hyperintensity | Restricted | 3m | Normal |
| 18 | Yes | Yes (splenium) | Normal | Hyperintensity | UA | 3m | Normal |
| 19 | Yes | Yes (splenium) | Bilateral posterior limbs of internal capsule | Hyperintensity | Restricted | 1.5m | UA |
| 20 | Yes | Yes | Bilateral posterior limbs of internal capsule | UA | Restricted | 2m | Elevated protein levels and white blood cells |
| 21 | Yes | Yes (splenium) | Bilateral parieto-occipital regions, posterior limbs of internal capsule and middle cerebellar peduncles | Hyperintensity | UA | 1.5y | Normal |
| 22 | Yes | Yes (splenium) | Normal | Hyperintensity | Restricted | 2m | Elevated protein levels (47mg/dl) and white blood cells (22/mm^3^) |
| 23 | Yes | Yes (splenium) | Normal | Hyperintensity | Restricted | 1m | Normal |
| 24 | Normal | Yes (splenium) | Bilateral parieto-occipital regions | Hyperintensity | Restricted | 3m | Elevated protein levels (78mg/dl) |
| 25 | Yes | Yes (splenium) | Normal | Hyperintensity | UA | 2m | Normal |
| 26 | Yes | Yes (splenium) | Normal | Hyperintensity | UA | 8w | UA |
| 27 | Normal | Splenium, genu | Bilateral parieto-occipital regions | Hyperintensity | UA | 3m | Normal |
| 28 | Yes | Yes (splenium) | Normal | Hyperintensity | Restricted | 101d | Elevated protein levels (46mg/dl) |
| 29 | Yes | Yes (splenium) | Normal | Hyperintensity | Restricted | 11d | Normal |
| 30 | Yes | Splenium, body, genu | Normal | Hyperintensity | Restricted | 3m | UA |
| 31 | Yes | Yes (splenium) | Normal | Hyperintensity | Restricted | 2y | Normal |
| 32 | Yes | Yes (splenium) | Bilateral parieto-occipital regions and posterior limbs of internal capsule | Hyperintensity | Restricted | 1y | Normal |
| 33 | Yes | Yes (splenium) | Normal | Hyperintensity | UA | 6m | Normal |
| 34 | Normal | Normal | Bilateral parietal lobe | Hyperintensity | UA | 1y | Normal |
| 35 | Yes | Yes | Normal | Hyperintensity | UA | 6w | UA |
| 36 | Yes | Yes | Normal | Hyperintensity | Restricted | 3m | UA |
| 38 | Yes | Splenium, body, genu | Bilateral occipital lobe | Hyperintensity | Restricted | 3m | Normal |
| 39 | Yes | Yes (splenium) | Bilateral frontal and parietal lobe | Hyperintensity | Restricted | 11w | Normal |
| 40 | Normal | Normal | Normal | Normal | Normal | None | UA |
| 41 | Yes | Normal | Bilateral parietal lobe and cerebellar peduncles | Hyperintensity | Restricted | 3m | Normal |
| 42 | Yes | Normal | Bilateral parietal lobe, unilateral cerebellar peduncle | Hyperintensity | UA | 6m | Normal |
| 43 | Normal | Yes (splenium) | Bilateral parieto-occipital regions | Hyperintensity | UA | 2m | Normal |
| 44 | Yes | Normal | Normal | Hyperintensity | UA | 1y | Elevated protein levels (60mg/dl) |
| 45 | Yes | Splenium, genu | Bilateral parietal lobe, posterior limbs of internal capsule and middle cerebellar peduncles | Hyperintensity | Restricted | 1m | UA |
| 46 | Yes | Normal | Normal | Hyperintensity | UA | UA | Normal |
| 47 | Yes | Normal | Normal | Hyperintensity | UA | UA | Normal |

MRI: Magnetic resonance imaging; CSF: cerebrospinal fluid; FLAIR, fluid-attenuated inversion recovery; DWI: diffusion-weighted imaging; m: month; d: day; y: year; w: week
