# Supplementary material for: Systematic review of CMTX1 patients with episodic neurological dysfunction
Source: Ann Clin Transl Neurol. 2020 Dec 12;8(1):213–23. doi: 10.1002/acn3.51271 (PMC7818278; doi:10.1002/acn3.51271)
Supplement: Supplementary file 4 — Table S4. The NCS results in CMTX1 patients with episodic neurological dysfunction [file ACN3-8-213-s004.docx]

Table S4. The NCS results in CMTX1 patients with episodic neurological dysfunction

| Patient  Number | Age for EMG | Median nerve | | | | | Ulnar nerve | | | | | Peroneal nerve | | | Tibial never | | | Sural nerve | |
| --- | --- | --- | --- | --- | --- | --- | --- | --- | --- | --- | --- | --- | --- | --- | --- | --- | --- | --- | --- |
|  |  | Distal latency  (ms) | CMAP amplitude  (mV) | MNCV  (m/s) | SNAP amplitude  (µV) | SNCV  (m/s) | Distal latency  (ms) | CMAP amplitude  (mV) | MNCV  (m/s) | SNAP amplitude  (µV) | SNCV  (m/s) | Distal latency  (ms) | CMAP amplitude  (mV) | MNCV  (m/s) | Distal latency  (ms) | CMAP amplitude  (mV) | MNCV  (m/s) | SNAP amplitude  (µV) | SNCV  (m/s) |
| 3 | 29 | 8.7 | 5.6 | 40.5 | 2.3 | 41.7 | 7.5 | 6.8 | 39.5 | 3.2 | 40.9 | 10.9 | 1.9 | 39.5 | 13.2 | 2.4 | 39.9 | 2.8 | 46.0 |
| 4 | 28 | 11.4 | 6.8 | 33.6 | 2.5 | 39.4 | 12.0 | 6.5 | 22.2 | UA | UA | UA | UA | UA | 16.9 | 3.8 | 33.0 | UA | UA |
| 9 | 17 | 4.5 | 4.2 | 27.0 | 2.0 | 38.0 | 3.5 | 2.7 | 36.0 | NR | NR | NR | NR | NR | 6.3 | 0.3 | 31.0 | UA | UA |
| 10 | 15 | 3.8 | 9.4 | 53.0 | 19.8 | 44.0 | 2.8 | 5.7 | 57.0 | 7.4 | 48.0 | 4.6 | 1.6 | 39.0 | 4.4 | 6.0 | 45.0 | UA | UA |
| 11 | 18 | 6.8 | 2.1 | 35.0 | NR | NR | 5.6 | 3.9 | 36.0 | NR | NR | NR | NR | NR | 10.1 | 0.3 | 24.0 | UA | UA |
| 14 | 22 | 6.9 | 0.8 | 31.3 | NR | UA | 4.9 | 4.0 | 33.6 | NR | UA | NR | NR | NR | UA | NR | NR | NR | UA |
| 16 | 22 | UA | 10.6 | 44.0 | 2.6 | 36.4 | UA | 10.4 | 45.2 | 2.9 | 33.9 | UA | 0.3 | 33.7 | UA | NR | NR | 4.3 | 28.7 |
| 18 | 13 | 6.6 | 4.4 | 37.0 | UA | UA | UA | UA | UA | UA | 40.0 | 5.8 | 0.3 | 26.0 | NR | 0.7 | 27.0 | UA | 39.0 |
| 20 | 15 | 4.2 | 1.7 | 40.0 | 3.2 | 33.0 | 2.7 | 8.9 | 39.0 | 1.8 | 36.0 | NR | NR | NR | NR | NR | NR | UA | UA |
| 22 | 12 | 5.2 | 1.7 | 26.2 | 20.5 | 42.9 | UA | UA | UA | UA | UA | NR | NR | NR | 6.8 | 1.9 | 28.1 | 2.8 | 29.9 |
| 23 | 18 | UA | UA | 39.0 | UA | 32.0 | UA | 38.0 | UA | UA | 36.0 | UA | UA | 33.0 | UA | UA | 38.0 | UA | 32.0 |
| 27 | 17 | 10.6 | 4.0 | 40.4 | 6.3 | UA | 8.7 | 4.0 | 36.8 | 6.2 | UA | 19.2 | 0.2 | 30.5 | 20.6 | 0.7 | 25.1 | NR | NR |
| 28 | 28 | 5.3 | 0.8 | 26.9 | NR | NR | 5.8 | 0.3 | 21.5 | NR | NR | UA | UA | UA | UA | UA | UA | UA | UA |
| 29 | 12 | 5.8 | 0.6 | 36.9 | 2.0 | 40.0 | 3.8 | 2.1 | 37.0 | 3.1 | 41.1 | 6.9 | 0.1 | 30.8 | 6.8 | 0.6 | 35.4 | 3.4 | 32.9 |
| 34 | 14 | 6.2 | 2.0 | 35.0 | 10.0 | 36.0 | UA | UA | UA | 5.0 | 34.0 | 4.2 | 2.5 | 32.0 | 6.0 | 4.7 | 33.0 | NR | NR |
| 46 | 16 | 4.6 | 0.9 | 32.0 | UA | UA | UA | UA | UA | UA | UA | 5.2 | 0.6 | 31.0 | UA | UA | UA | 5.0 | 32.0 |
| 47 | 17 | 4.2 | 3.6 | 35.0 | UA | UA | UA | UA | UA | UA | UA | 7.1 | 2.7 | 37.0 | UA | UA | UA | 3.0 | 31.0 |
| mean ± SD | 18.4±  5.5 | 6.3±  2.3 | 3.7±  3.0 | 36.0±  6.7 | 7.1±  7.3 | 38.3±  4.0 | 5.7±  3.0 | 7.8±  9.9 | 36.7±  9.7 | 4.2±  2.0 | 38.7±  4.8 | 8.0±  5.0 | 1.1±  1.0 | 33.3±  4.2 | 10.1±  5.6 | 2.1±  2.0 | 32.7±  6.5 | 3.6±  0.9 | 33.9±  5.8 |

NR: No response; UA: Unavailable; SD: standard deviation; CMAP: compound muscle action potential; MNCV: motor nerve conduction velocity; SNAP: sensory nerve action potential; SNCV: sensory nerve conduction velocity
